# Analytical correlation between overall subjective satisfaction and criterion-based evaluation in continuing medical education: A cross-sectional study of associated factors

**DOI:** 10.1371/journal.pone.0349900

**Published:** 2026-05-27

**Authors:** Hela Ghali, Asma Ben Cheikh, Sana Bhiri, Latifa Lassoued, Imed Chouchen

**Affiliations:** 1 Department of Preventive and Community Medicine, Sahloul University Hospital, Sousse, Tunisia; 2 Faculty of Medicine of Sousse, University of Sousse, Sousse, Tunisia; 3 Department of Obstetrics and Gynecology, Farhat Hached University Hospital, Sousse, Tunisia; 4 Medical Intensive Care Unit, Farhat Hached University Hospital, Sousse, Tunisia; University of Huelva: Universidad de Huelva, SPAIN

## Abstract

**Background:**

Continuing Medical Education (CME) is essential for updating the competencies of healthcare professionals. As part of a quality improvement initiative, the CPD committee at the Faculty of Medicine of Sousse evaluated the 2021–2022 programs. The study aimed to assess learner satisfaction by distinguishing overall subjective satisfaction from criterion-based evaluation (based on specific quality criteria like resources and pedagogy). This approach identifies whether a positive general feeling masks structural deficiencies in course design.

**Methods:**

We conducted a cross-sectional study over a five-week period using a structured evaluation grid distributed via Google Forms. The evaluation covered all three phases of the CME process (before, during, and after the course). For each criterion, participants selected one of four options ranging from “unsatisfied” to “satisfied.” Criterion-based evaluation was assessed via an overall rating from 1 to 5, with a score ≥4 indicating satisfaction. Correlation between overall subjective satisfaction and criterion-based evaluation was analyzed.

**Results:**

A total of 268 responses were collected. Women predominated (73.1%). The mean age was 36 ± 8 years, and most participants practiced in urban areas (91%) and at the tertiary care level (55.6%). The overall mean satisfaction score was 3.8 ± 1.02, with 69.4% of participants classified as satisfied. Overall subjective satisfaction was significantly associated with criterion-based evaluation across all criteria. In addition, overall subjective satisfaction was associated with age (p = 0.007), years in current practice (p = 0.043), academic affiliation (p = 0.008), specialty (p = 0.019), and type of CME (p = 0.003).

**Conclusion:**

Learner satisfaction with CME activities was correlated with factors such as specialty, academic affiliation, experience, age, and CME format. These findings support recommendations to tailor CME content to participant profiles, emphasize practical training, ensure timely delivery of resources, and improve advance communication of schedules to enhance program effectiveness.

## Introduction

Evaluation processes are central to educational quality assurance, as they systematically gather, organize, and interpret information about the design, delivery, and outcomes of programs [1]. In medical education, evaluation serves multiple purposes: assessing the feasibility of interventions before implementation, monitoring fidelity during delivery, measuring impacts at learner and patient levels, and informing decisions on resource allocation [2]. These processes are vital to support continuous improvement, guide strategic planning, and ensure accountability to learners, institutions, and society. Continuing Medical Education (CME) remains a core strategy to maintain and improve physicians’ competence throughout their careers, aiming to ensure that health professionals keep pace with rapidly evolving medical knowledge, digital technologies, and standards of care [3,4].

Despite decades of investment, the impact of CME on healthcare delivery remains inconsistent. There is growing consensus that CME must evolve from passive content delivery to approaches that actively enhance physician competence and improve patient health outcomes. Evaluation is an essential component of these efforts; among its various dimensions, learner satisfaction is often the first and most frequently measured serving as an initial indicator of program quality [5,6].

Satisfaction in educational settings can be approached from distinct yet complementary perspectives. Overall subjective satisfaction typically reflects learners’ overall subjective appraisal of the training experience, whereas criterion-based evaluation relies on structured assessments aligned with predefined domains or criteria. This distinction is consistent with broader educational evaluation frameworks that differentiate self-declared (subjective) profiles from framework-based (objective) profiles derived from expected performance standards. For instance, Alvarez-Icaza et al. [7] emphasized that combining subjective and objective perspectives provides a more comprehensive understanding of student development and learning outcomes. Furthermore, criteria-based approaches have been highlighted as a way to strengthen the transparency and interpretability of evaluation measures by aligning them with explicit objectives and predefined standards rather than relying solely on global impressions [8]. Together, these perspectives support the dual use of perceived and measured satisfaction, allowing for both an overall learner-centered judgment and a domain-specific, actionable assessment of the learning experience.

The rationale for focusing on learner satisfaction is further grounded in its role as a prerequisite for higher-level educational outcomes. The manuscript utilizes established training evaluation models, such as the outcomes framework developed by Moore [9] for CME evaluation. According to Moore, satisfaction assessment (Level 2) corresponds to a foundational level of a multi-level scale, serving as the basis upon which subsequent levels, such as knowledge acquisition and performance change are built. While satisfaction alone does not guarantee clinical impact, it is a critical indicator of the program’s perceived value and learner engagement. In the absence of frequent research-based evaluations, particularly in resource-constrained settings, assessing satisfaction becomes a strategic necessity. It provides essential diagnostic feedback on the feasibility and acceptability of training, ensuring the educational environment is optimized to support the long-term transfer of skills into clinical practice.

Understanding the relationship between overall subjective satisfaction and criterion-based evaluation is therefore critical for effective CME evaluation. If a strong correlation exists, a simple global rating might serve as a valid proxy for more detailed assessments; conversely, if these measures diverge, relying solely on overall subjective satisfaction could be misleading. Yet, few studies have explicitly examined this relationship in low- and middle-income countries (LMICs) like Tunisia. Therefore, this study was designed to assess overall satisfaction, examine the correlation between overall subjective satisfaction and criterion-based evaluation, and identify sociodemographic and professional factors associated with these outcomes.

## Methods

### Study design

This cross-sectional study was conducted among all learners who took part in CME during the 2021–2022 academic year at the Faculty of Medicine of Sousse, as part of the commitment of the Continuing Professional Development (CPD) committee at the Faculty of Medicine of Sousse to improving CME quality. This committee refers to the ongoing process of maintaining, enhancing, and updating professional knowledge and skills throughout a practitioner’s career.

In Tunisia, the system of Continuing Medical Education (CME) is primarily governed and delivered by the four national Faculties of Medicine. While professional medical associations and the private sector contribute to educational offerings, the university remains the main institutional guarantor of quality and formal certification. The participants in these programs are predominantly healthcare professionals, including general practitioners and specialists, who have completed their initial medical education and are currently engaged in clinical practice. The CME structure typically consists of two main formats: ‘structured’ curricula, such as the *Certificates of Complementary Studies*, which are year-long programs involving theoretical and practical assessments, and master’s degrees which are two-years-long programs involving theoretical and practical assessments. This study specifically evaluates the programs organized under the aegis of the Faculty of Medicine of Sousse, reflecting its role in maintaining the professional competence of physicians in the central region of the country.

### Study population

The survey was aimed at all learners who took part in any CME activities conducted during the 2021–2022 academic year at the Faculty of Medicine of Sousse, including both certificate and master’s level programs. The study included 268 participants, the majority of whom were licensed health professionals, including physicians in general practice, medical and surgical specialists, and a small proportion of pharmacists and dentists.

### Data collection

Data were collected anonymously via an online questionnaire posted on google forms.

This study was conducted as a quality improvement initiative by the Continuing Professional Development (CPD) committee at the Faculty of Medicine of Sousse. The development process was informed by a review of educational evaluation literature and aligned with the WFME Global Standards for Quality Improvement in CPD [10], ensuring that the criteria covered the full educational cycle. The content was further refined based on the collective professional expertise of the researchers and committee members to ensure relevance to the local Tunisian CME context. The questionnaire was written in French and pre-tested with a small group of clinicians to ensure the clarity of the items and the flow of the digital interface. The internal reliability of the evaluation grid was assessed using Cronbach’s alpha coefficient, yielding a value of 0.84, which demonstrates high internal consistency.

After gathering all the electronic mail, including the survey link and invitation to participate addresses, we attempted to contact potential CME participants by sending an electronic mail including the survey link and invitation to participate, inviting the receiving learner to respond and briefly introducing the purpose of the study. Two secretaries from the faculty dean’s office handled these electronic mails, including the survey link and invitation to participate. These electronic mails, including the survey link and invitation, have been sent twice. Each sending attempt was scheduled to be followed by one week of rest before another invitation was sent.; the total duration of the electronic mail, including the survey link and invitation to participate, was five weeks

Data collection concluded two months after the initial invitation email was distributed. No financial incentives or promotional activities were offered to participants to encourage their response.

### Study tool

The survey tool was a structured electronic questionnaire (Google Forms), written in French and pre-tested.

The evaluation covered all three phases of the course, i.e., before starting, during and at the end of the course.

For each criterion, the learner chose one of four propositions on a Likert-like scale. To facilitate statistical analysis, we have grouped the two categories “not at all” and “rather no” into a single “disagree” category, and the other two categories into “agree”.

To assess overall subjective satisfaction, at the end, the learner was asked to give an overall assessment of the CME activity, followed by assigning an overall mark from 1 to 5, with 5 meaning the highest mark and 1 the lowest in terms of performance. Participants are classified as satisfied if they give an overall rating of 4 or higher.

### The online survey consisted of three sections

- The first one aimed to collect sociodemographic information, including:

- Age, gender, grade (health professional or not) according to the world health organization definition for health professional [11], medical specialty, institution, number of years in practice,
- Governorate and practice area which can be urban area (municipality, high population density, importance of non-agricultural activities...), rural area (“Imada”, low population density, importance of agricultural activities...) or semi-rural area (transitional environment, such as delegation...)
- **Current level of practice (organization of care system) which includes the first** level (basic health center, district hospital, rural maternity hospital, intermediate center, maternal and child protection center, tuberculosis control center, etc.), second level (Regional Hospital, National Social Security Fund), tertiary care level (university hospitals) (University Hospital), or other (Ministry, Regional Directorate, Authority...)

- The second section of the surveys addressed the type of CME participated in: certificate of complementary studies or master’s degrees with a title specified
- The last section of the survey included a questionnaire aimed at evaluating the three phases of the course. A total of 20 criteria were evaluated, grouped as follows:
- At the start of the CME (3 criteria)
- During CME (9 criteria)
- At the end of the CME (8 criteria)

This three-stage distinction was designed to align with the WFME Global Standards for CPD [12] evaluation and to minimize recall bias by guiding participants through the specific sequence of the educational experience. Although these phases were aggregated into a composite ‘measured satisfaction’ score for statistical analysis, to provide a stable and comprehensive metric for correlation with perceived satisfaction, the initial separation ensured that all dimensions of the training cycle were systematically represented and evaluated.

The last question was an open-ended one, allowing learners to offer their suggestions for improving the learning they have undergone as part of their continuing medical education in the 2021–2022 academic year.

### Data analysis

Statistical analysis was performed using SPSS ver. 25.0 (IBM, Chicago, IL, USA).

We determined the criterion-based evaluation based on these criteria. Thus, those who answered the criterion with “not at all” or “rather no” were considered as “not satisfied” and those who answered with “rather yes” or “completely agree” were considered as “satisfied”.

Data are represented as numbers and percentages for ordinal variables and mean ±standard deviation or median and interquartile range in square brackets [IQR] for continuous variables.

The Kolmogorov-Smirnov test, a nonparametric test used to assess whether a sample follows a specified distribution, such as normality [13], was performed to evaluate variables distribution.

To assess the correlation between overall subjective satisfaction and criterion-based evaluation, we used Chi-squared test, which was used to determine whether there is a significant association between two variables in contingency tables. Overall subjective satisfaction refers to the participants’ overall rating of the CME on a 1–5 scale, while criterion-based evaluation refers to their responses to the 20 specific criteria evaluated across the three phases of the CME. The strength of association was measured via the Phi coefficient. The Phi coefficient is a measure of effect size ranging from 0 to 1 (for positive associations), where a value of 0 indicates no relationship and 1 indicates perfect association. Based on standard statistical conventions, the strength of the association was interpreted as follows:

- **0.10 < Phi < 0.30**: Weak association;
- **0.30 < Phi < 0.50**: Moderate association;
- **Phi > 0.50**: Strong association.

To account for multiple comparisons across the 20 satisfaction items, a Bonferroni correction was applied (p < 0.0025).

To assess factors associated with overall subjective satisfaction, the Chi-squared test was used to compare categorical variables, and Student t-test (A parametric test used to compare the means of two groups) or the U- MannWhitney test (A nonparametric alternative to the t-test, used when comparing two independent groups without assuming normal distribution) to compare quantitative variables [14]. Significant p-value was set at 0.05.

### Ethics approval and consent to participate

The protocol for this study was submitted to the Ethics Committee of the Faculty of Medicine of Sousse, Tunisia. The committee issued a formal statement (Ref: CEFMSo_0056) concluding that formal ethical approval was not required, as the study constitutes an evaluation of educational programs and professional practices aimed at quality improvement.

Nevertheless, the research was conducted in accordance with the ethical principles of the Declaration of Helsinki. All participants were informed of the study’s objectives and the voluntary nature of their participation. Data were collected anonymously through a secure digital platform, and electronic informed consent was obtained from all learners prior to their participation. No personal identifying information was recorded, ensuring the total confidentiality of the respondents.

## Results

### Participants’ characteristics

Overall, we received 268 responses. Women predominated (73.1% vs. 26.9%).

The mean age was 36 ± 8 years. The majority of our study population has completed a certificate of Most of our population worked in an urban area (91%; n = 244). In 55.6% of cases, the level of practice was tertiary care level (university hospitals).

The Institution of first-degree was the faculty of medicine of Sousse in half the cases.

The source of information for registration was either the Faculty of Medicine of Sousse website (57.1%), or a colleague (47.8%).

A detailed description of participants’ characteristics is presented in Table 1.

**Table 1:** Demographic profile of study population, Faculty of medicine of Sousse, academic year 2021–2022.

Characteristics		Statistics N(%)
**Gender n (%)**	Male	72 (26.9)
	Female	196 (73.1)
**Age n (%)**	<30 years	74 (27.6)
	≥ 30–40 years	121 (45.1)
	≥ 40–50 years	52 (19.4)
	50 or above	21 (7.8)
**Function**	Health Professionnal	255 (95.1)
	Not Health Professionnal	13 (4.9)
**Institution of first-degree n (%)**	Faculty of Medicine	134 (50)
	Other	134 (50)
**Governorate of the current setting n (%)**	Tunis	58 (21.7)
	Bizerte	1 (0.4)
	Jendouba	2 (0.7)
	Kef	1 (0.4)
	Nabeul	5 (1.9)
	Zaghouan	3 (1.1)
	Sousse	118 (44)
	Mahdia	11 (1.2)
	Monastir	16 (6)
	Siliana	4 (1.5)
	Kairouan	16 (6)
	Kasserine	4 (1.5)
	Sidi Bouzid	2 (0.7)
	Sfax	20 (7.5)
	Mednine	1 (0.4)
	Foreign	5 (1.9)
**Area of the current setting n (%)**	Urban	244 (91)
	Rural	5 (1.9)
	Semi rural	19 (7.1)
**Current level of practice n (%)**	First level	62 (23.1)
	Second level	22 (8.2)
	Third level	149 (55.6)
	Other	35 (13.1)
**Seniority in the current practice in years (median [IQR])**		5 [4–93 –10]
**Specialty of health professionals (n = 255)**	General medicine	35 (13.1)
	Family Medicine	54 (20.1)
	Surgical specialties	29 (10.8)
	Medical specialties	72 (26.9)
	Fondamental specialities	44 (16.3)
	Pharmacy	5 (1.9)
	Dentistry	6 (2.2)
	Other	23 (8.6)
**Type of CME n (%)**	Certificate of complementary studies	132 (49.3)
	Master’s degree	136 (50.7)

### Evaluation of CME

The two types of CME were certificates of complementary studies (n = 132; 49.3%) and master’s degree (n = 136; 50.7%).

The mean overall subjective satisfaction of the CME was 3.8 ± 1.02. The proportion of learners rated as satisfied with the course was 69.4%.

Satisfaction levels were significantly higher among those who had taken a certificate of complementary studies (78%) compared to those with a master’s degree (61%; p = 0.003).

### Correlation between overall subjective satisfaction and criterion-based evaluation

Participants were classified as satisfied if they gave an overall rating of 4 or higher. For each evaluated criterion, we studied the correlation between overall subjective satisfaction and criterion-based evaluation.

Overall subjective satisfaction was significantly associated with criterion-based evaluation for all criteria.

The association between global overall subjective satisfaction and the 20 specific dimensions of criterion-based evaluation was analyzed using the Phi coefficient. All items demonstrated statistically significant positive associations (p < 0.001). The strength of these associations was predominantly moderate, with Phi values ranging from 0.205 to 0.503. The strongest correlation was observed for the alignment of the learning process with the stated program (phi = 0.503), followed by the respect for the teaching program (phi = 0.449) and the fulfillment of the role as a medical expert (phi = 0.449). These results indicate that while logistical factors (e.g., registration) are linked to satisfaction, pedagogical consistency and professional relevance are the primary drivers of high learner endorsement.

Results are summarized in Table 2.

**Table 2:** Correlation between overall subjective satisfaction and criterion-based evaluation.

Overall subjective satisfaction Criterion-based evaluation		Satisfied n (%)	Not satisfied n (%)	p-value	Phi-value
**At the start of the CME course**					
**Registration procedure at the start of the course has been made available**	Satisfied	163 (87.6)	58 (70.7)	0.001	0.205
	Not satisfied	23 (12.4)	24 (29.3)		
**A syllabus describing the course (statement of objectives/learning outcomes, learning methods and assessment strategy) has been made available**	Satisfied	168 (92.3)	53 (67.9)	0.000	0.313
	Not satisfied	14 (7.7)	25 (32.1)		
**Registration fees are adapted to the course**	Satisfied	152 (88.4)	48 (69.6)	0.001	0.226
	Not satisfied	20 (11.6)	21 (30.4)		
**During CME**					
**The learning process is in line with the stated program**	Satisfied	177 (95.2)	44 (53.7)	0.000	0.503
	Not satisfied	9 (4.8)	38 (46.3)		
**Resources/citations (textbooks, sections,** **articles, videos...) were appropriate for the learner’s learning process**	Satisfied	170 (91.4)	45 (54.9)	0.000	0.423
	Not satisfied	16 (8.6)	37 (45.1)		
**Learning was active and learner-centered**	Satisfied	176 (94.6)	54 (65.9)	0.000	0.380
	Not satisfied	10 (5.4)	28 (34.1)		
**Teachers were motivating and mastered the content and flow of learning**	Satisfied	179 (96.2)	55 (67.1)	0.000	0.404
	Not satisfied	7 (3.8)	27 (32.9)		
**Teaching program respected**	Satisfied	176 (94.6)	48 (58.5)	0.000	0.449
	Not satisfied	10 (5.4)	34 (41.5)		
**Timetable respected**	Satisfied	168 (90.3)	46 (56.1)	0.000	0.393
	Not satisfied	18 (9.7)	36 (43.9)		
**Dates of training sessions announced on time**	Satisfied	163 (87.6)	50 (61.0)	0.000	0.304
	Not satisfied	23 (12.4)	32 (39.0)		
**Assessments or exams (ongoing and final) related to the skills required during the learning process**	Satisfied	177 (95.2)	57 (69.5)	0.000	0.355
	Not satisfied	9 (4.8)	25 (30.5)		
**Date of theory test announced on time**	Satisfied	173 (93.0)	54 (65.9)	0.000	0.348
	Not satisfied	13 (7.0)	28 (34.1)		
**At the end of the course**					
**This training has enabled me to fulfil my role as a medical expert**	Satisfied	177 (95.2)	49 (59.8)	0.000	0.449
	Not satisfied	9 (4.8)	33 (40.2)		
**This training has enabled me to fulfil my role as a communicator with patients and their families**	Satisfied	157 (84.4)	44 (53.7)	0.000	0.327
	Not satisfied	29 (15.6)	38 (46.3)		
**This training has enabled me to fulfill my role as a collaborator with other healthcare professionals**	Satisfied	172 (92.5)	52 (63.4)	0.000	0.361
	Not satisfied	14 (7.5)	30 (36.6)		
**This training has enabled me to fulfill my role as a leader who collaborates with other clinicians, administrators, researchers...**	Satisfied	150 (80.6)	35 (42.7)	0.000	0.378
	Not satisfied	36 (19.4)	47 (57.3)		
**This training has enabled me to fulfil my role as a Health Advocate, working with communities or patient populations to improve health**	Satisfied	152 (81.7)	34 (41.5)	0.000	0.403
	Not satisfied	34 (18.3)	48 (58.5)		
**This training has helped me as a researcher**	Satisfied	139 (74.7)	27 (32.9)	0.000	0.397
	Not satisfied	47 (25.3)	55 (67.1)		
**This training has served me as a Professional committed to the health and well-being of patients and society**	Satisfied	170 (91.4)	56 (68.3)	0.000	0.293
	Not satisfied	16 (8.6)	26 (31.7)		
**This training has served me to acquire the competence of the learned physician by ensuring the development of new skills**	Satisfied	177 (95.2)	53 (64.6)	0.000	0.403
	Not satisfied	9 (4.8)	29 (35.4)		

### Factors associated with overall subjective satisfaction

**Overall subjective satisfaction** was significantly associated with several participant characteristics.

Participants who were older reported higher satisfaction; the mean age of satisfied respondents was 36.98 ± 8.49 years compared to 33.99 ± 7.10 years among those not satisfied (OR = 1.05 per year increase, 95% CI [1.01–1.08], p = 0.007). Type of CME was also significant: 78% of those with a certificate of complementary studies were satisfied versus 61% of those with a master’s degree (p = 0.003). Participants affiliated with the Faculty of Medicine of Sousse had lower satisfaction (61.9%) compared to those from other institutions (76.9%, OR = 0.49 [0.28–0.83], p = 0.008). Greater seniority in practice was modestly associated with higher satisfaction (median 5 vs. 4 years; OR = 1.04 [1.001–1.096], p = 0.043). Specialty category also mattered: satisfaction was particularly high among surgical specialties (93.1%) compared to general practice (60.7%), with an overall p-value of 0.019. No significant differences in satisfaction were observed by gender, area of practice setting, or current level of practice. Results of analysis of associated factors are summarized in Table 3.

**Table 3:** Factors associated with overall subjective satisfaction.

Factor		Satisfied n (%)	Not satisfied n (%)	OR [CI_95%_]	p-value
**Gender**	Male	52 (72.2)	20 (27.8)	–	0.544
	Female	134 (68.4)	62 (31.6)		
**Age (mean±SD)**		36.98 ± 8.49	33.99 ± 7.10	1.05 [1.01-1.08]	0.007
**Type of CME**	Certificate of complementary studies	103 (78)	29 (22)	0.44 [0.2 – 0.75]	0.003
	Master’s degree	83 (61)	53 (39)		
**Function**	Health professional	174 (68.2)	81 (31.8)	–	0.066
	Not health professional	12 (92.3)	1 (7.7)		
**Institution**	Faculty of medicine of Sousse	83 (61.9)	51 (38.1)	0.49 [0.28-0.83]	0.008
	Other	103 (76.9)	31 (23.1)		
**Seniority in the current practice in years (median [IQR])**		5 [5–114 –12]	4 [3 –8]	1.04 [1.001-1.096]	0.043
**Specialty category**	General	54 (60.7)	35 (39.3)	–	0.019
	Medicine	46 (63.9)	26 (36.1)		
	Surgery	27 (93.1)	2 (6.9)		
	Fundamental	32 (72.7)	12 (27.3)		
	Other	15 (71.4)	6 (28.6)		
**Area of the current setting**	Urban	170 (69.7)	74 (30.3)	–	0.373
	Rural	2 (40)	3(60)		
	Semi rural	14 (73.7)	5 (26.3)		
**Current level of practice**	First level	37 (59.7)	25 (40.3)	–	0.260
	Second level	16 (72.7)	6 (27.3)		
	Third level	106 (71.1)	43 (28.9)		
	Other	27 (77.1)	8 (22.9)		

## Discussion

In health professions education, diverse evaluation methods are used, mainly focusing on outcome evaluation rather than program evaluation. These evaluations aim to collect and interpret information about program development, implementation, and outcomes [15,16]. Such evaluations are essential for refining continuing medical education (CME) content and delivery, assessing both short- and long-term impacts, and informing resource allocation. Measuring learner satisfaction is a critical component of CME evaluation, representing the initial level in Kirkpatrick’s model [17], and reflecting the extent to which participants’ expectations are met [9,18]. This model categorizes outcomes into four distinct tiers: Level 1 (Reaction), which assesses the degree to which participants find the training favorable, engaging, and relevant; Level 2 (Learning), measuring the acquisition of intended knowledge or skills; Level 3 (Behavior), evaluating the application of these skills to clinical practice; and Level 4 (Results), which examines the long-term impact on patient outcomes and organizational goals.

A significant contribution of this study lies in its methodological critique of how learner satisfaction is captured. Traditionally, CME evaluation has relied on "one-off" global retrospective questions, a single post-course rating that asks participants to summarize their entire experience. While administratively efficient, these one-dimensional metrics are increasingly viewed as insufficient for genuine quality assurance [19]. Our findings suggest that a multifaceted assessment approach, stratified into distinct chronological phases (Before, During, and After), provides a more robust and objective "Criterion-Based Evaluation" score.

This longitudinal-style structure is essential to mitigate the "halo effect" or recency bias, where an exceptionally engaging final session might lead a learner to overlook significant logistical failures during the planning or registration phase [20]. By disaggregating the educational cycle, we align our evaluation with the higher levels of Moore’s expanded framework, which advocates for moving beyond simple participation to assessing the structural and instructional components that drive competence [9,21].

Furthermore, the novelty of this multifaceted approach resides in its diagnostic granularity. While an overall subjective satisfaction score indicates if a program succeeded, a phase-based framework explains where it succeeded or failed. For instance, our data allowed us to distinguish between pedagogical satisfaction (content delivery) and organizational satisfaction (logistics). This level of detail is a prerequisite for the WFME Global Standards for Quality Improvement, which demand that CPD providers utilize evaluation data for specific, iterative program refinement [12].

Ultimately, the implications for future CME evaluation design are clear: moving away from simplistic, one-off surveys toward multidimensional grids is necessary to capture the complexity of the adult learning experience. This shift ensures that satisfaction is treated not as a monolith, but as a dynamic construct influenced by the entire educational trajectory, from initial infrastructure to immediate utility [9,22].

Our study, conducted as a cross-sectional survey over five weeks, utilized an evaluation grid distributed via Google Forms among all learners who participated in CME during the 2021-2022 academic year at the Faculty of Medicine of Sousse, Tunisia. The study aimed to gauge learner satisfaction with CME programs and identify factors associated with their overall subjective satisfaction.

Our findings align with most studies reporting the results of CME evaluation in terms of impact on physician knowledge, performance, and patient outcomes. Assessing learner satisfaction essentially constitutes an evaluation of teaching by learners, a concept developed in Anglo-Saxon higher education institutions several decades ago. However, the slow diffusion of assessment practices beyond Anglo-Saxon countries can be attributed to cultural resistance to the notion of teaching assessment by students, among other factors [23].

Consistent with prior research [24], our study found generally positive perceptions of the CME program among learners, an endorsement that aligns with findings by Odayappan et al.[25] regarding the high acceptability of structured clinical updates. However, the identified predictors of satisfaction, specifically age and seniority, suggest a nuanced developmental trajectory in medical education. Our finding that more experienced practitioners report higher satisfaction levels resonates with the work of Cook et al. [26], which indicates that mid-to-late career physicians often find greater value in formal activities that validate their established clinical expertise. This trend reflects the distinction between "expert" learners, who derive satisfaction from integrating new evidence into existing mental models, and "novice" learners, who may require more foundational, structured support to feel equally engaged [9].

Furthermore, the higher satisfaction observed among surgical specialists compared to general practitioners highlights a potential pedagogical gap, suggesting that current CME formats may be better optimized for technical, discipline-specific content than for the broad, multi-faceted needs of primary care, a discrepancy also noted in recent European studies on multi-specialty platforms [27].

Our findings highlight the complex role of institutional affiliation in shaping learner perception. The significant influence of institutional affiliation, particularly the higher satisfaction linked to Academic Health Centers, supports the observations of Rayburn et al. [28] regarding the role of medical schools as 'trusted hubs' for continuous learning. However, a nuanced trend emerged where certain internal affiliates reported lower satisfaction compared to external participants. This disparity may stem from higher baseline expectations or 'logistical fatigue' among staff members who are more familiar with institutional processes. This suggests that while medical schools act as trusted anchors, they also face more critical appraisal from their own stakeholders. Collectively, these findings underscore that moving beyond global satisfaction metrics is essential to addressing the diverse learning styles and organizational cultures that shape the perceived value of CME in both local and international contexts. By utilizing a criterion-based evaluation approach, CPD committees can identify specific pedagogical gaps, such as the need for more advanced 'medical expert' content for internal specialists, thereby ensuring the faculty remains a competitive center of excellence.

The type of CME activity also influenced satisfaction ratings, highlighting the importance of tailoring educational formats to learner preferences and needs. Furthermore, institutional affiliation played a significant role: participants affiliated with the Faculty of Medicine of Sousse reported lower satisfaction compared to those from other institutions, who exhibited a notably higher satisfaction rate. This difference was statistically significant, suggesting that institutional factors such as resource availability, organizational support, or educational culture may impact learner experiences and satisfaction.

Together, these findings underscore the multifaceted nature of CME satisfaction and point to potential areas for targeted interventions, including customization of content to different learner demographics and institutional contexts to maximize engagement and perceived benefit.

These findings resonate with those of Jayas et al. [24], who found high satisfaction levels among CME participants, particularly among those in surgery specialties, with academic affiliations and participation in professional meetings and journal-based CME being significant factors associated with satisfaction [24].

Moreover, the academic year 2021-2022 was characterized by the COVID-19 pandemic, which disrupted CME activity, particularly for foreign learners and face-to-face teaching, despite previous preferences for in-person CME offerings among health professionals [29,30]. Recent studies on physicians' perceptions of virtual CME alternatives prompted by the pandemic have highlighted preferences for hybrid formats and emphasized the complementary role of virtual offerings alongside traditional in-person offerings [25,27,31,32].

Our observations regarding age align with the notion that mid-career physicians (40–49 years old) may face challenges in work-life balance, suggesting a need for highly flexible, on-demand CME approaches for this demographic [26]. Furthermore, specialty category and academic affiliation emerged as professional attributes independently associated with satisfaction with CME programs, suggesting tailored approaches for specific specialties and highlighting the potential benefits of academic affiliations in enhancing CME satisfaction.

We have identified two professional attributes that are independently correlated with our findings: specialty category and academic affiliation. Our insights into specific specialties may be of interest to organizations and professional development programs tailored to those specialties. Notably, our results showed that physicians in surgical specialties reported a significantly higher satisfaction rate compared to general practitioners, indicating that CME approaches tailored to surgical disciplines may be particularly well-received.

This discrepancy may be explained by the inherent differences in program delivery variability between these fields. Surgical CME is often characterized by a "competency-based" instructional design, focusing on structured, technical, and procedural content that aligns well with the goal-oriented learning styles of surgeons. In such programs, the delivery is typically more standardized and the outcomes more immediate, which often correlates with higher perceived value.

Conversely, the lower satisfaction observed among general practitioners suggests a challenge in delivery alignment. Primary care CME often addresses "ill-defined" or psychosocial clinical scenarios that are harder to standardize. When these complex topics are delivered through rigid, didactic formats, there is often a mismatch between the multifaceted nature of general practice and the instructional method [33]. This suggests that the variability in how content is delivered, ranging from technical workshops to broad clinical updates, is a primary driver of the satisfaction gap between specialties. Moving forward, CME providers should adopt specialty-specific delivery models that account for the unique cognitive and procedural demands of each discipline, rather than employing a "one-size-fits-all" approach [34].

We noted a higher likelihood of satisfaction with the CME program among individuals affiliated with Academic Health Centers (AHCs) or teaching hospitals. This suggests that the 2021–2022 period, a phase of recovery and adaptation following the COVID-19 pandemic, likely served as a catalyst for our findings regarding institutional affiliation and resource access. During the pandemic, AHCs emerged as critical hubs for rapid information dissemination, and our findings may reflect the superior digital infrastructure and support systems these institutions maintained during the shift to hybrid CME formats [24].

As emphasized by Rayburn et al. [28], there are expanding opportunities to harness the esteemed status of medical schools as hubs for ongoing learning. The transition of traditional face-to-face activities into virtual events at academic institutions presented a unique avenue for broadening physician access during the COVID-19 landscape. However, the pandemic also highlighted a distinct accessibility gap for younger physicians. This demographic’s lower satisfaction may be explained by the high requirements for flexibility and interactivity in a post-COVID landscape [35]; if digital delivery remained didactic rather than interactive, it likely failed to meet the expectations of digitally native learners. Leveraging the trusted status of academic institutions as value centers remains a promising strategy to bridge this gap, provided that the digital infrastructure is used to foster engagement rather than just passive content delivery.

### Recommendations

- **Timely provision of learning resources:** Develop a detailed distribution schedule and online access systems.
- **Align examinations with taught material:** Regularly review exam content and gather feedback to ensure relevance.
- **Emphasize practical components:** Reassess curriculum to incorporate more hands-on activities and real-world examples.
- **Improve organization of sessions:** Provide clear agendas and advance notification of session topics and dates.
- **Balance class distribution:** Ensure manageable workloads and timely communication of session schedules.

Our study has several methodological limitations that necessitate a cautious and objective interpretation of the results. First, the study is subject to selection bias stemming from voluntary participation via digital platforms; this may have led to a ‘volunteer effect’ where respondents with more polarized views or higher digital engagement are overrepresented, potentially skewing the findings toward more favorable outcomes. Regarding the response rate, while 268 responses provided a substantial dataset, the lack of demographic data on non-participants prevents a definitive assessment of non-response bias, which is a common constraint in electronic survey-based research. Second, a measurement bias is inherent in the reliance on self-reported, subjective questionnaires, which are susceptible to social desirability and the ‘halo effect’. While the evaluation instrument demonstrated high internal consistency (Cronbach’s alpha = 0.84) and the significant correlations (Phi coefficients up to 0.503) suggest a structured perception of quality, these associations may also reflect elements of circular analysis. In this context, a learner’s general positive institutional impression may have influenced their ratings across specific pedagogical criteria, rather than each criterion being evaluated independently. Furthermore, as a primarily descriptive study focused on Kirkpatrick Level 1 (Reaction), these metrics represent a prerequisite for learner engagement rather than a direct measure of knowledge acquisition or long-term changes in clinical practice. Finally, the generalizability of these findings is limited by the specific geographic and institutional context of the Tunisian medical education system, and the transition to digital tools may have introduced a digital access bias against less tech-savvy practitioners, further impacting the diversity of the captured learner experience.

## Conclusion

This study demonstrates that while learners were generally satisfied with their CME program, with higher satisfaction independently associated with older age, greater seniority, surgical specialty, and academic affiliation, the true value of these findings lies in the methodological framework used to uncover them. Our analysis highlights the critical importance of moving beyond ‘one-off’ retrospective surveys toward multifaceted assessment designs. By disaggregating the educational experience into chronological phases, we provide a more granular diagnostic tool that can distinguish between pedagogical success and logistical friction, offering a more robust model for future CME evaluation.

Furthermore, the observed differences in satisfaction across specialties suggest that program delivery variability and instructional design significantly influence perceived value. Future CME offerings should not only be tailored to physician demographics but must also align delivery methods, ranging from structured, procedural training for surgeons to complex, case-based learning for general practitioners, to the specific cognitive needs of each discipline. Further research is warranted to explore the causal relationship between these multifaceted assessment scores and actual clinical practice changes. Ultimately, adopting such nuanced evaluation and delivery frameworks is essential to ensure that CME remains a high-impact tool for improving the quality of patient care.

## Supporting information

S1 Data Final dataset english. (XLSX)
